# Associations of genetic variants of miRNA coding regions with pulmonary tuberculosis risk in China

**DOI:** 10.1017/S0950268821002004

**Published:** 2021-08-31

**Authors:** Mingwu Zhang, Zhengwei Liu, Yelei Zhu, Songhua Chen, Bin Chen, Xiaomeng Wang

**Affiliations:** Zhejiang Provincial Center for Disease Control and Prevention, Binsheng Road, Binjiang District, Hangzhou City, Zhejiang Province, 310051, China

**Keywords:** Haplotype, microRNA, pulmonary tuberculosis, risk, single-nucleotide polymorphisms

## Abstract

Tuberculosis (TB) is the leading cause of death caused by single pathogenic microorganism, *Mycobacterium tuberculosis* (MTB). The study aims to explore the associations of microRNA (miRNA) single-nucleotide polymorphisms (SNPs) with pulmonary TB (PTB) risk. A population-based case−control study was conducted, and 168 newly diagnosed smear-positive PTB cases and 251 non-TB controls were recruited. SNPs located within miR-27a (rs895819), miR-423 (rs6505162), miR-196a-2 (rs11614913), miR-146a (rs2910164), miR-618 (rs2682818) were selected and MassARRAY^®^ MALDI-TOF System was employed for genotyping. SPSS19.0 was adopted for statistical analysis, non-conditional logistic regression was performed. Odds ratios (ORs) and 95% confidence intervals (95% CIs) were computed to estimate the associations. Associations of haplotypes with PTB risk were performed with online tool. Rs895819 CT/CC genotype was associated with reduced PTB risk among female population (OR = 0.45, 95% CI: 0.23–0.98), *P* = 0.045. Haplotypes (combined with rs895819, rs2682818, rs2910164, rs6505162 and rs11614913) TCCCT, TAGCC, CCCCC, CCGCT and TCGAT were associated with reduced PTB risk and the ORs were 0.67 (95% CI: 0.45–0.99), 0.49 (0.25–0.94), 0.34 (95% CI: 0.14–0.81), 0.22 (95% CI: 0.06–0.84) and 0.24 (95% CI: 0.07–0.79), respectively; while the haplotypes of TAGCT, CCCCT, CACCT and TCCAT were associated with increased PTB risk, and the ORs were 3.63 (95% CI: 1.54–8.55), 2.20 (95% CI: 1.00–4.86), 3.90 (95% CI: 1.47–10.36) and 2.95 (95% CI: 1.09–7.99), respectively. Rs895819 CT/CC genotype was associated with reduced female PTB risk and haplotype TCCCT, TAGCC, CCCCC, CCGCT and TCGAT were associated with reduced PTB risk, while TAGCT, CCCCT, CACCT and TCCAT were associated with increased risk.

## Introduction

In 2018 about 10.0 million people fell ill with tuberculosis (TB), while 1.2 million human immunodeficiency virus (HIV)-negative individuals and an additional 251 000 HIV-positive individuals died of TB worldwide [1]. Although that year an estimated 1.7 billion people harboured *Mycobacterium tuberculosis* (MTB) infections, only a relatively small proportion (5–10%) of them would potentially develop active TB during their lifetimes.

The progression from MTB infection to TB disease is influenced by multiple factors, including undernutrition, smoking, alcohol consumption, etc. [2]. In addition, host molecular regulatory mechanisms, such as gene-expression regulation by microRNAs (miRNAs), may also be involved in disease progression. As a class of non-coding ribonucleic acids (RNAs)，miRNAs play key roles in regulating post-transcriptional gene expression during numerous physiological and pathological processes, including MTB infection and TB progression to active disease [3]. During MTB infection, which lies at the root of active TB disease in humans, downregulated expression of miRNAs homo sapiens (hsa)-miR-29a and hsa-miR-15b has been reported [4]. By contrast, hsa-miR-576-5p, hsa-miR-500 and hsa-miR-155 expression have been shown to be upregulated during latent tuberculosis infection (LTBI) [3]. Intriguingly, miRNA effects have been demonstrated during different stages of TB progression; for example, Beibei Fu *et al*. found that miR-325-3p was upregulated after MTB infection then demonstrated that miR-325-deficient mice exhibited MTB resistance [5]. These findings collectively shed light on the immune escape pathway used by MTB, whereby MTB infection directly targeted LNX1 expression that led to anti-apoptotic STAT3 signalling that ultimately promoted intracellular MTB survival [5]. Meanwhile, another study showed that hsa-miR-29a-3p, hsa-miR-155-5p and hsa-miR-361-5p were upregulated during active tuberculosis (ATB) as compared to their expression in healthy subjects; of these miRNAs, hsa-miR-29a-3p, and hsa-miR-361-5p were found to be upregulated during ATB as compared to their expression during LTBI [4]. Notably, MiR-143 and miR-365 have been pinpointed as key regulators of MTB infection, with roles in MTB-infected macrophages that influence regulation of host cell c-Maf, Bach-1 and Elmo-1 activities [6]. In another study, a mimic of miR-708-5p enhanced intracellular mycobacterial survival during MTB infection, while miR-708-5p downregulation suppressed MTB survival [7]. Meanwhile, another study found that upregulation of miR-579 induced macrophage cytotoxicity that countered MTB infection by targeting the cPWWP2A-miR-579 axis to ultimately protect human macrophages from MTB infection [8].

Genetic variants of expressed miRNA regions have been implicated in their biogenic effects, and these effects appear to influence or alter downstream biological processes to cause disease [9]. For example, the miR-146a single-nucleotide polymorphism (SNP) rs2910164 has been shown to be associated with ischaemic stroke incidence and prognosis [10–12]. Meanwhile, other studies have revealed that miRNAs participate in MTB infection and TB incidence, although studies showing relationships between miRNA SNPs and TB incidence risk have been limited or have remained obscure. To explore the potential effects of SNPs located within miRNA coding regions on PTB ris, the SNPs reported to be associated immune-regulation-related diseases in previous studies in recent years were selected. In addition, biological functions of the selected miRNAs with genetic variants were analysed through their putative targeted genes. Altogether six SNPs located within miR-27a (rs895819), miR-423 (rs6505162), miR-196a-2 (rs11614913), miR-146a (rs2910164), miR-618 (rs2682818) and miR-34a (rs35301225) were recruited, however no mutation was detected in miR-34a coding region in our study. So in the present study, the SNPs at rs895819, rs6505162, rs11614913, rs2910164 and rs2682818 were selected and their associations with TB incidence were analysed.

## Materials and methods

### Ethics statement

The study was approved by the ethics committee of the Zhejiang Provincial Center for Disease Control and Prevention. Written informed consent was obtained from each participant prior to participation in the study.

### Study population

A population-based case−control study was conducted in Jiangshan and Changshan counties in Zhejiang Province. In the controlled study, 168 newly diagnosed smear-positive pulmonary TB (PTB) patients were enrolled from 1st January 2016 to 30th June 2017 along with 251 control subjects recruited from the same hospitals. Control subjects included clinically diagnosed patients suffering from non-TB illnesses or healthy individuals. All recruited controls verified they had no history of TB.

All participants were asked to complete a structured questionnaire and provide personal demographic information and lifestyle-related characteristics, such as smoking, drinking, etc. Other information collected from each study subject included health-related factors and disease diagnosis and treatment histories.

### Genotyping

In the present study, a MassARRAY^®^ MALDI-TOF System (US, Sequenom, Inc.) was employed for genotyping. During genotyping, three primers for each SNP were designed and primer sequences are shown in Table 1. The genotyping protocol was implemented as described below.

1. Polymerase chain reaction (PCR)

**Table 1.** Primers designed for the miRNA SNPs genotyping

SNP NO.	^a^Primers	Sequences (5′−3′)
rs895819	P1	ACGTTGGATGGGAACTTAGCCACTGTGAAC
	P2	ACGTTGGATGAGGGCTTAGCTGCTTGTGAG
	P3	CCCCCCTGTGAACACGACTTGG
rs6505162	P1	ACGTTGGATGTCCAAAAGCTCGGTCTGAGG
	P2	ACGTTGGATGACTGTCTCTCTTCACACTGC
	P3	TAGAAACTCAAGCGCGGG
rs11614913	P1	ACGTTGGATGCTGATCTGTGGCTTAGGTAG
	P2	ACGTTGGATGTCGACGAAAACCGACTGATG
	P3	CGGCAACAAGAAACTG
rs2910164	P1	ACGTTGGATGGAACTGAATTCCATGGGTTG
	P2	ACGTTGGATGCCACGATGACAGAGATATCC
	P3	GGTTTTGTCAGTGTCAGACCT
rs2682818	P1	ACGTTGGATGTTCCATGAGCTGCTGATGAA
	P2	ACGTTGGATGACGTACATGCAGTAGCTCAG
	P3	CAGGAGACAAGCAGG

a P1 and P2 were adopted for PCR amplification, and P3 for single base extension.

Each PCR amplification reaction was prepared in a total volume of 5 μl and contained 1 μl (30 ng) DNA template, 0.625 μl of 10 × PCR buffer (15 mM MgCl_2_, Qiagen, Germany), 0.325 μl of MgCl_2_ (25 mM, Qiagen, Germany), 1 μl of dNTPs (2.5 mM each, TaKaRa, Dalian, China), 0.1 μl of Hotstar Taq (0.5 U, Qiagen, Germany), 1 μl of diluted each primers and 0.95 μl of ddH_2_O. PCR cycling parameters were as follows: DNA denaturation at 94 °C for 10 min followed by 35 cycles of 94 °C for 20 s, 56 °C for 20 s, and 72 °C for 60 s followed by a final extension step of 72 °C for 3 min and storage at 4 °C until needed.

1. SPA typing reaction preparation

For SPA typing, 2 μl of SPA reaction buffer was added into each PCR plate well then each reaction was incubated using the following parameters: 37 °C for 40 min followed by 85 °C for 5 min. Completed reactions were maintained at 4 °C until needed.

1. Single base extension

For single base extension, 2 μl of iPlex reaction reagent (supplied by SEQUENOM as a solution containing 0.2 μl of 10 × iPlex buffer, 0.2 μl of iPlex termination mix, 0.804 μl of diluted primer and 0.041 μl of iPlex enzyme). PCR reaction parameters was set as follows: one cycle at 94 °C for 30 s followed by 40 cycles of annealing at 94 °C for 5 s then 5 cycles of 52 °C for 5 s and 80 °C for 5 s then a final extension step at 72 °C for 3 min. Completed reactions were stored at 4 °C until needed.

1. Reaction product purification and data analysis

Reaction products were purified and spotted onto sample plates according to standard protocols. Draft data were generated then processed using TYPER genotyping analysis software.

10% of randomly selected DNA samples were rechecked using the same method.

### Statistical analysis

In the present study, SPSS software version 19.0 was used for statistical analysis. Chi-square tests were used to compare distributions of demographic factors, including gender, occupation, family income, nationality, smoking and alcohol use. Means ages of PTB patient and control groups were compared using independent-*t* tests. Non-conditional logistic regression was employed, with inclusion of age, sex, smoking and alcohol use in regression models as covariates followed by computing of adjusted odds ratios (ORs) and 95% confidence intervals (95% CIs). Haplotype analyses for all five SNPs were performed using online software (http://analysis.bio-x.cn/myAnalysis.php); haplotypes with frequencies less than 0.03 were excluded from the final analysis. ORs and 95% CIs for each tested haplotype were compared to all other haplotypes in order to estimate the contribution of each haplotype to PTB risk. A two-sided *P* value less than 0.05 was considered significant.

## Results

### Distributions of demographic characteristics

Comparisons of demographic characteristics between groups of PTB cases and controls were performed and are shown in Table 2. Proportion of those aged ⩾60 years in the control group was lower than that in the case group (53.4% *vs.* 64.1%) *P* = 0.030). A higher proportion of alcohol users was found in the control group as compared to the case group (37.8% *vs.* 24.2%), *P* = 0.005. The proportion of subjects with annual household income per capita ⩾10 000 RMB in the control group was higher than the proportion in the case group (67.5% *vs.* 54.2%, respectively), *P* = 0.014. No significant intergroup differences were detected for sex, occupation, smoking and nationality.

**Table 2.** Demographic characteristics of recruited populations

Variables	Control (*n*, %)	Case (*n*, %)	*t*/*χ*^2^	*P* value
Age			4.690	0.030
＜60 years	117(46.6)	60(35.9)		
⩾60 years	134(53.4)	107(64.1)		
Sex			0.039	0.844
Male	162(64.5)	110(65.5)		
Female	89(35.5)	58(34.5)		
Occupation			1.858	0.173
Farmers	200(80.6)	123(75.0)		
Others	48(19.4)	41(25.0)		
Smoking			0.046	0.830
Yes	95(37.8)	58(38.9)		
No	156(62.2)	91(61.1)		
Drinking			7.828	0.005
Yes	94(37.8)	36(24.2)		
No	155(62.2)	113(75.8)		
Nationalities			-	0.567^a^
Chinese han	250(99.6)	166(98.8)		
Others	1(0.4)	2(1.2)		
Family income			5.985	0.014
⩾ 10 000 RMB	137(67.5)	71(54.2)		
< 10 000 RMB	66(32.5)	60(45.8)		

a Computed with Fisher's exact test.

### Association of miRNA SNPs with PTB risk

From Table 3, it can be seen that none of the SNPs selected for study, including miR-27a (rs895819), miR-423 (rs6505162), miR-196a-2 (rs11614913), miR-146a (rs2910164) and miR-618 (rs2682818), were associated with PTB risk in the overall study population. However, after stratification by sex, associations of male and female populations with PTB incidence were determined and are shown in Table 4. A significant association was detected between the rs895819CT/CC genotype and TB risk for the female population, as reflected by OR = 0.45 (95% CI: 0.23–0.98) (Table 4), while no significant association was detected between this genotype and PTB incidence for the male population. Meanwhile, no significant associations were observed between other SNPs and PTB risk for either the male or female population.

**Table 3.** Associations of the miRNA SNPs with PTB risk

Variables	Control (*n*, %)	Case (*n*, %)	^a^Adjusted ORs(95% CIs)	*P* value
rs895819 (miR-27a)				
TT	140(57.8)	113(67.7)	Reference	
CT	90(37.2)	48(28.7)	0.69(0.44–1.07)	0.095
CC	12(5.0)	6(3.6%)	0.67(0.24–1.88)	0.441
CT/CC	102(42.2)	54(32.3)	0.70(0.45–1.09)	0.110
rs6505162 (miR-423)				
CC	174(70.7)	110(65.5)	Reference	
CA	67(27.3)	50(29.7)	1.32(0.84–2.08)	0.228
AA	5(2.0)	8(4.8)	2.80(0.88–8.96)	0.082
AA/AC	72(29.3)	58(34.5)	1.37(0.87–2.14)	0.171
rs11614913 (miR-196a-2)				
TT	66(26.8)	52(31.0)	Reference	
CT	135(54.9)	77(45.8)	0.76(0.48–1.22)	0.256
CC	45(18.3)	39(23.2)	1.09(0.61–1.95)	0.768
CT/CC	180(73.2)	116(69.0)	0.81(0.51–1.28)	0.369
rs2910164 (miR-146a)				
CC	93(38.2)	61(36.7)	Reference	
CG	109(44.9)	86(51.8)	1.17(0.75–1.82)	0.488
GG	41(16.9)	19(11.5)	0.65(0.34–1.25)	0.199
CG/GG	150(61.8)	105(63.3)	0.98(0.63–1.51)	0.910
rs2682818 (miR-618)				
CC	117(48.4)	81(48.5)	Reference	
CA	107(44.2)	72(43.1)	0.96(0.63–1.47)	0.865
AA	18(7.4)	14(8.4)	1.04(0.48–2.23)	0.925
CA/AA	125(51.6)	86(51.5)	0.95(0.62–1.45)	0.810

a Adjusted for age, sex, smoking and drinking.

**Table 4.** Associations of miRNA SNPs with PTB risk among male and female population

Male					Female			
Variables	Control (*n*, %)	Case (*n*, %)	^a^Adjusted ORs (95% CIs)	*P* value	Control (*n*, %)	Case (*n*, %)	^a^Adjusted ORs (95% CIs)	*P* value
rs895819 (miR-27a)								
TT	92(58.6)	71(64.5)	Reference		48(56.5)	42(73.7)	Reference	
CT	57(36.4)	36(32.7)	0.96(0.54–1.69)	0.882	33(38.8)	12(21.1)	0.40(0.17–0.96)	0.039
CC	8(5.0)	3(2.8)	0.70(0.17–2.87)	0.615	4(4.7)	3(5.2)	0.79(0.17–3.82)	0.772
CT/CC	65(41.4)	39(35.5)	0.93(0.54–1.60)	0.785	37(43.5)	15(26.3)	0.45(0.23–0.98)	0.045
rs6505162 (miR-423)								
CC	108(68.4)	71(64.5)	Reference		66(75.0)	39(67.2)	Reference	
CA	47(29.7)	33(30.0)	1.22(0.69–2.17)	0.489	20(22.7)	17(29.3)	1.17(0.52–2.64)	0.700
AA	3(1.9)	6(5.5)	3.77(0.87–16.32)	0.076	2(2.3)	2(3.5)	1.71(0.23–12.85)	0.603
AA/AC	50(31.6)	39(35.5)	1.38(0.80–2.38)	0.253	22(25.0)	19(32.8)	1.22(0.56–2.66)	0.615
rs11614913 (miR-196a-2)								
TT	37(23.4)	34(30.9)	Reference		29(33.0)	18(31.0)	Reference	
CT	93(58.9)	47(42.7)	0.55(0.29–1.04)	0.067	42(47.7)	30(51.7)	1.05(0.48–2.32)	0.899
CC	28(17.7)	29(26.4)	1.23(0.58–2.60)	0.586	17(19.3)	10(17.3)	1.05(0.37–3.00)	0.926
CT/CC	121(76.6)	76(69.1)	0.71(0.39–1.28)	0.257	59(67.0)	40(69.0)	1.05(0.50–2.23)	0.894
rs2910164 (miR-146a)								
CC	60(38.2)	35(32.1)	Reference		33(38.4)	26(45.6)	Reference	
CG	73(46.5)	62(56.9)	1.23(0.69–2.18)	0.486	36(41.9)	24(42.1)	0.78(0.36–1.71)	0.536
GG	24(15.3)	12(11.0)	0.83(0.34–2.00)	0.671	17(19.7)	7(12.3)	0.57(0.20–1.68)	0.310
CG/GG	97(61.8)	74(67.9)	1.13(0.65–1.97)	0.655	53(61.6)	31(54.4)	0.72(0.35–1.49)	0.379
rs2682818 (miR-618)								
CC	76(48.4)	53(48.6)	Reference		41(48.2)	28(48.3)	Reference	
CA	70(44.6)	48(44.0)	0.94(0.54–1.64)	0.840	37(43.5)	24(41.4)	0.85(0.40–1.80)	0.669
AA	11(7.0)	8(7.4)	1.11(0.37–3.31)	0.848	7(8.3)	6(10.3)	1.08(0.29–3.95)	0.911
CA/AA	81(51.6)	56(51.4)	0.97(0.56–1.65)	0.896	44(51.8)	30(51.7)	0.88(0.43–1.80)	0.735

a Adjusted for age, smoking and drinking.

After stratification by age, associations between PTB risk and genotype for those aged <60 years and ⩾60 were determined and are shown in Table 5. No significant associations between PTB risk and genotype were detected among those aged <60 years. Conversely, in those aged ⩾60 years (Table 5), a significant association was detected between the rs895819CT genotype and increased PTB risk (OR = 0.53, 95% CI: 0.28–0.99), although no significant association was found between genotype rs895819CT/CC and reduced PTB risk (OR = 0.59, 95% CI: 0.32–1.06). However, a marginally significant association was found between the rs6505162AA/AC genotype and increased PTB risk for those aged ⩾60 years (OR = 1.69, 95% CI: 0.93–3.06), *P* = 0.083.

**Table 5.** Associations of miRNA SNPs with PTB risk among different age groups

Aged ＜60 years					Aged ⩾60 years			
Variables	Control (*n*, %)	Case (*n*, %)	^a^Adjusted ORs (95% CIs)	*P* value	Control (*n*, %)	Case (*n*, %)	^a^Adjusted ORs (95% CIs)	*P* value
rs895819 (miR-27a)								
TT	59(52.7)	34(57.6)	Reference		81(62.3)	78(72,9)	Reference	
CT	46(41.1)	24(40.7)	0.90(0.44–1.82)	0.760	44(33.9)	24(22.4)	0.53(0.28–0.99)	0.047
CC	7(6.2)	1(1.7)	0.28(0.03–2.49)	0.256	5(3.8)	5(4.7)	1.07(0.29–3.97)	0.925
CT/CC	53(47.3)	25(42.4)	0.82(0.41–1.63)	0.565	49(37.7)	29(27.1)	0.59(0.32–1.06)	0.075
rs6505162 (miR-423)								
CC	76(66.1)	39(65.0)	Reference		98(74.8)	70(65.4)	Reference	
CA	36(31.3)	18(30.0)	0.97(0.46–2.02)	0.930	31(23.7)	32(29.9)	1.53(0.83–2.83)	0.176
AA	3(2.6)	3(5.0)	1.78(0.33–9.59)	0.503	2(1.5)	5(4.7)	4.44(0.79–24.95)	0.090
AA/AC	39(33.9)	21(35.0)	1.04(0.52–2.10)	0.906	33(25.2)	37(34.6)	1.69(0.93–3.06)	0.083
rs11614913 (miR-196a-2)								
TT	32(27.8)	18(36.0)	Reference		34(26.0)	33(30.8)	Reference	
CT	67(58.3)	26(52.0)	0.72(0.32–1.62)	0.432	68(51.9)	51(47.7)	0.71(0.38–1.33)	0.283
CC	16(13.9)	16(32.0)	2.19(0.84–5.71)	0.109	29(22.1)	23(21.5)	0.72(0.34–1.57)	0.412
CT/CC	83(62.2)	42(84.0)	1.00(0.47–2.12)	0.999	97(74.0)	74(69.2)	0.71(0.39-)1.29	0.263
rs2910164 (miR-146a)								
CC	45(39.5)	21(36.2)	Reference		48(37.2)	39(36.4)	Reference	
CG	50(43.9)	31(53.4)	1.01(0.49–2.10)	0.978	59(45.7)	55(51.4)	1.16(0.64–2.11)	0.620
GG	19(16.6)	6(10.4)	0.72(0.24–2.11)	0.545	22(17.1)	13(12.2)	0.69(0.29–1.65)	0.403
CG/GG	69(60.5)	37(63.8)	0.93(0.47–1.85)	0.839	81(62.8)	68(63.6)	1.03(0.59–1.82)	0.914
rs2682818 (miR-618)								
CC	57(50.4)	32(54.2)	Reference		60(46.5)	48(44.9)	Reference	
CA	50(44.2)	24(40.7)	0.88(0.44–1.77)	0.724	57(44.2)	48(44.9)	0.94(0.53–1.67)	0.825
AA	6(5.4)	3(5.1)	1.19(0.27–5.25)	0.821	12(9.3)	11(10.2)	0.99(0.37–2.67)	0.985
CA/AA	56(49.6)	27(45.8)	0.91(0.46–1.79)	0.790	69(53.5)	59(55.1)	0.95(0.54–1.64)	0.843

a Adjusted for sex, smoking and drinking.

### Associations of haplotypes with PTB risk

Haplotypes with frequencies less than 0.03 were excluded from the final analysis. Associations between PTB risk and the remaining haplotypes of SNPs rs895819, rs2682818, rs2910164, rs6505162 and rs11614913 are summarised in Table 6.

**Table 6.** Associations of haplotypes with PTB risk

^a^Haplotype	Case (*n*,%)	Control (*n*,%)	Chi^2^	Fisher' *P*	Pearson' *P*	ORs (95% CI)
TCCCT	43(13.1)	85(17.8)	3.945	0.047	0.047	0.67 (0.45–0.99)
TCCCC	48(14.5)	59(12.4)	0.557	0.456	0.455	1.17 (0.77–1.77)
TCGCT	39(11.8)	45(9.5)	0.909	0.340	0.340	1.25 (0.79–1.97)
TCGCC	25(7.7)	21(4.4)	3.647	0.056	0.056	1.78 (0.98–3.25)
TAGCC	13(3.9)	35(7.4)	4.827	0.028	0.028	0.49 (0.25–0.94)
TACCT	17(5.0)	33(6.9)	1.438	0.230	0.230	0.69 (0.37–1.27)
TACCC	12(3.7)	18(3.8)	0.009	0.925	0.925	0.97 (0.46–2.03)
TAGCT	19(5.7)	8(1.6)	9.858	0.002	0.002	3.63 (1.54–8.55)
CCCCC	6(1.9)	26(5.4)	6.475	0.011	0.011	0.34 (0.14–0.81)
TCCAC	11(3.2)	19(4.0)	0.439	0.507	0.507	0.77 (0.36–1.67)
CCCCT	16(4.8)	11(2.2)	4.006	0.045	0.045	2.20 (1.00–4.86)
CACCT	15(4.6)	6(1.2)	8.551	0.003	0.003	3.90 (1.47–10.36)
TACAC	11(3.3)	7(1.6)	2.586	0.108	0.108	2.13 (0.83–5.45)
TCCAT	12(3.6)	6(1.2)	4.955	0.026	0.026	2.95 (1.09–7.99)
CCGCT	3(0.8)	16(3.3)	5.888	0.015	0.015	0.22 (0.06–0.84)
TCGAT	3(1.0)	18(3.9)	6.479	0.011	0.011	0.24 (0.07–0.79)

a Combined with SNPs of rs895819, rs2682818, rs2910164, rs6505162 and rs11614913 in turn.

Of all included haplotypes, TCCCT had the highest frequency and was significantly associated with reduced PTB risk (OR = 0.67, 95% CI: 0.45–0.99), *P* = 0.047. Among the other haplotypes, similar significant associations were detected for TAGCC, CCCCC, CCGCT and TCGAT, with ORs of 0.49 (95% CI: 0.25–0.94), 0.34 (95% CI: 0.14–0.81), 0.22 (95% CI: 0.06–0.84) and 0.24 (95% CI: 0.07–0.79), respectively. By contrast, haplotypes TAGCT, CCCCT, CACCT and TCCAT were associated with increased TB risk, with ORs of 3.63 (1.54–8.55), 2.20 (1.00–4.86), 3.90 (1.47–10.36) and 2.95 (1.09–7.99), respectively.

## Discussion

In this study, SNPs located within expressed miRNA regions and their associations with PTB risk were analysed. After stratification of results according to sex, we observed a significant association between rs895819 and overall TB risk of the female population. Several haplotypes of five SNPs were found to be significantly associated with PTB incidence.

MiRNAs, a class of micro-molecules, play key roles in gene-expression regulation and in multiple biological and pathological processes, including embryonic development, tumorigenesis, immunoregulation and inflammation [13]. When considered together, SNPs located within expressed miRNA regions and binding sites may be useful for predicting an individual' future risk of contracting infectious and non-infectious diseases. To date, SNPs and miRNA sequence-based variations have been applied to the prediction of tumour-associated diseases, asthma, TB, cardiovascular and cerebrovascular diseases (among others) in recent decades [14, 15]. For example, the SNP located within the 3′ untranslated region (3′ UTR) of the phosphatase and tensin homologue (*PTEN*) gene has been found to be associated with cervical cancer risk [16]. Another miRNA, miR-27a, has also been found to play key roles in regulating gene expression and proliferation of ovarian cancer and breast cancer cells [17, 18]. In the present study, one SNP located within the expressed sequence of miR-27a was found to be associated with decreased PTB incidence in the female population, but not in the male population [19]. Intriguingly, Wang *et al*. found that downregulation of miR-27a expression in MTB-infected THP-1 cells led to enhanced expression of interferon-*γ* (IFN-*γ*), interleukin-beta (IL-*β*), IL-6 and tumour necrosis factor-alpha (TNF-*α*); this enhanced cytokine expression was abolished by cell transfection with miR-27a mimics that also led to downregulated target gene IRAK4 expression [19]. Taken together, these findings offer clues that will likely enhance our understanding of potential molecular mechanisms associated with TB disease incidence. Indeed, progress has already been made toward this goal in another study that verified that endoplasmic reticulum-located Ca(2+) transporter CACNA2D3 was another miR-27a target gene [20]. When miR-27a activity was suppressed, downregulation of Ca(2+) signalling and subsequent inhibition of autophagosome formation occurred that promoted greater intracellular MTB survival [20]. Meanwhile, another SNP located within the expressed region of miR-27a was found to be associated with increased colorectal cancer (CRC) risk and reduced breast cancer risk [21–23]. These studies indicate that SNPs located within the expressed region of miR-27a were functional SNPs with putative roles in gene regulation that consequently influenced the occurrences of certain diseases.

Our study revealed taht the SNP of rs895819 located within miR-27a coding region had an effect on PTB risk in female but not in male population, and the difference might be affected by the potential functions that decided by its putative target genes. Just as it was reported in the previous study by Mushtaq Ahmad *et al*., the SNP effects the size of the terminal loop in the precursor miRNA of miR-27a and then modify the common breast cancer risk, which was very common in female population [24]. And in ovarian cancer cell lines, another common female cancer cell line, the F-box and WD-40 domain protein 7 (FBXW7), a classical tumour suppressor, was found directly targeted by miR-27a and its translation was suppressed by miR-27a [25]. Furthermore, Zhang *et al*. found that miR-27a promoted the progression of ovarian cancer cells and induced the process of EMT via the Wnt/*β*-catenin signalling pathway through inhibition of FOXO1[26]. While Li *et al*. found that Inositol polyphosphate-1-phosphatase (INPP1) up-regulation regulated by miR-27a contributed to the growth, migration and invasion of human cervical cancer [27]. From these studies, we realised that the SNP located within miR-27a was associated with some female cancer risk, while the similar association with non-female cancer was rare, so the molecular regulation mechanism of miR-27a was relatively complex. Just like the findings in our present study, the potential molecular mechanisms need to be clarified by further studies. Other miRNAs have been linked to human diseases as well. For example, results of one study implicated involvement of both miR-146 and miR-29 in post-transcriptional regulation of the renalase gene, a gene which contributes to observed inter-individual variations of cardiometabolic traits [28]. In other studies, the SNP located within miR-146a (rs2910164) was confirmed to be associated with increased coronary artery disease (CAD) risk [29] and reduced gastric cancer risk [30]. Although these studies also indicated that the SNP located within miR-146a was a functional SNP, this SNP did not appear to be associated with PTB incidence in the current study and thus was not found to affect TB risk. Similarly, other studies have shown that SNPs located within miR-196a-2 were associated with oral squamous cell carcinoma (OSCC) survival, but not with breast cancer risk [21] or colorectal cancer (CRC) risk [31], aligning with results of this study showing no association of miR-196a-2 with PTB risk.

Another miRNA linked to human disease, miR-423, was shown in one study to activate NF-*κβ* and promote breast cancer invasion [32] while miR-423-5p was shown in another study to be upregulated during active TB, highlighting its potential as a PTB biomarker [33]. Meanwhile, the SNP (rs6505162) located within the expressed miR-423 region was associated with lower risks of gastrointestinal cancer, CRC and lung cancer, but was not associated with higher risks of oesophageal cancer, breast cancer or gastric cancer [34]. Moreover, previous studies had identified miR-423 as a key factor associated with TB incidence that exerted its functional effects via gene regulation, although here this SNP was not found to be significantly associated with PTB incidence [33].

Yet another miRNA liked to human disease, miR-618, has been reported to suppress gastric cancer metastasis by downregulating transforming-growth factor beta 2 (TGF-2*β*) expression and to inhibit prostate cancer cell migration and invasion by targeting FOXP2 and downregulating TGF-*β* [35]. Meanwhile, results of a meta-analysis study revealed that SNP rs2682818, located within the expressed sequence region of miR-618, was not associated with overall cancer risk, but was specifically associated with breast cancer risk [36]. However, another study revealed that rs2682818 was linked to reduced CRC risk [37].

Haplotype analysis is a method that evaluates various combinations of multiple genetic variants to find haplotypes that are useful for predicting disease risk. In the present study, combinations of various haplotypes were evaluated to determine their associations with PTB risk. Ultimately, a strategy for predicting future disease incidence that was based on multiple SNPs was found to be superior to one based on a single SNP.

In order to discover potential SNP-disease associations for different age groups, stratification by age was conducted. Notably, SNPs with marginal levels of statistical significance were detected within expressed sequences of miR-423 and miR-27a for subjects aged ⩾60 years, but not for subjects aged <60 years. Thus, we speculate that significant SNP correlations with PTB disease may were not detected for some age groups, due to their statistically inadequate sample sizes. As a result, although the present study provided some useful information, further studies based on larger sample sizes are needed to verify the conclusions of this study and to discover SNPs with statistically stronger correlations with PTB risk.

Based on the results of this study, we conclude that rs895819 was associated with reduced female PTB incidence, while this SNP together with rs6505162 may be useful for predicting PTB risk in patients aged ⩾60 years of age. Analysis of haplotypes with regard to SNPs rs895819, rs2682818, rs2910164, rs6505162 and rs11614913 may find useful markers for effective prediction of PTB risk.

## Abbreviations

PTB: pulmonary tuberculosis; MTB: *Mycobacterium tuberculosis*, miRNAs: microRNAs; SNP: single-nucleotide polymorphism; LTBI: latent tuberculosis infection; ATB: active tuberculosis; TB: tuberculosis; PCR: polymerase chain reaction; ORs: odds ratios; 95% CIs: 95% confidence intervals; 3′ UTR: 3′ untranslated region; PTEN: phosphatase and tensin homologue; IFN-*γ*: interferon-*γ*; IL-*β*: interleukin-beta; TNF-*α*: tumour necrosis factor-alpha; OSCC: oral squamous cell carcinoma; CRC: colorectal cancer; TGF-beta: transforming growth factor-beta

## Declaration

A questionnaire was developed for this study and never published elsewhere.

## Data

Supporting data can be acquired via correspondence with the author.
